# Supplementary material for: Bioinformatics analysis of microarray data to identify the candidate biomarkers of lung adenocarcinoma
Source: PeerJ. 2019 Jul 10;7:e7313. doi: 10.7717/peerj.7313 (PMC6626531; doi:10.7717/peerj.7313)
Supplement: Supplemental Information 1 [file peerj-07-7313-s001.docx]

| **Supplemental table 1 The top 15 Gene Ontology terms of upregulated DEGs** | | | | | |
| --- | --- | --- | --- | --- | --- |
| **Category** | | **Term** | **Count** | **p.adjust(FDR)** | |
| BP | extracellular matrix organization | | 11 | | 0.000108 |
| BP | extracellular structure organization | | 11 | | 0.00023 |
| BP | extracellular matrix disassembly | | 5 | | 0.004435 |
| BP | collagen catabolic process | | 4 | | 0.006452 |
| BP | oligosaccharide biosynthetic process | | 3 | | 0.012706 |
| CC | extracellular matrix | | 11 | | 0.000166 |
| CC | collagen-containing extracellular matrix | | 8 | | 0.001552 |
| CC | collagen trimer | | 4 | | 0.011185 |
| CC | fibrillar collagen trimer | | 2 | | 0.015365 |
| CC | banded collagen fibril | | 2 | | 0.015365 |
| MF | serine-type endopeptidase activity | | 8 | | 0.000873 |
| MF | serine-type peptidase activity | | 8 | | 0.000873 |
| MF | serine hydrolase activity | | 8 | | 0.000873 |
| MF | glycosaminoglycan binding | | 7 | | 0.001358 |
| MF | extracellular matrix structural constituent | | 6 | | 0.001361 |
| BP: biological process; CC: cellular component; MF: molecular function | | | | | |
