# Supplementary material for: Bioinformatics analysis of microarray data to identify the candidate biomarkers of lung adenocarcinoma
Source: PeerJ. 2019 Jul 10;7:e7313. doi: 10.7717/peerj.7313 (PMC6626531; doi:10.7717/peerj.7313)
Supplement: Supplemental Information 2 [file peerj-07-7313-s002.docx]

| **Supplemental table 2 Top 15 Gene Ontology terms of downregulated DEGs** | | | | | |
| --- | --- | --- | --- | --- | --- |
| **Category** | | **Term** | **Count** | **p.adjust(FDR)** | |
| BP | response to corticosteroid | | 18 | | 4.12E-08 |
| BP | response to glucocorticoid | | 17 | | 4.12E-08 |
| BP | cell-substrate adhesion | | 23 | | 3.42E-07 |
| BP | response to alcohol | | 19 | | 4.08E-07 |
| BP | response to peptide | | 27 | | 4.08E-07 |
| CC | extracellular matrix | | 33 | | 8.88E-12 |
| CC | collagen-containing extracellular matrix | | 27 | | 1.30E-11 |
| CC | membrane raft | | 16 | | 0.000322 |
| CC | membrane microdomain | | 16 | | 0.000322 |
| CC | membrane region | | 16 | | 0.000359 |
| MF | growth factor binding | | 14 | | 4.66E-06 |
| MF | extracellular matrix structural constituent | | 13 | | 8.20E-05 |
| MF | scavenger receptor activity | | 8 | | 8.46E-05 |
| MF | glycosaminoglycan binding | | 15 | | 8.46E-05 |
| MF | amyloid-beta binding | | 8 | | 0.000221 |

BP: biological process; CC: cellular component; MF: molecular function
